# Comparative study of photocatalysis with bulk and nanosheet graphitic carbon nitrides enhanced with silver

**DOI:** 10.1038/s41598-024-62291-w

**Published:** 2024-05-20

**Authors:** Monika Michalska, Jiri Pavlovsky, Grazyna Simha Martynkova, Gabriela Kratosova, Viktoria Hornok, Peter B. Nagy, Vlastimil Novak, Tamas Szabo

**Affiliations:** 1 Department of Chemistry and Physico-Chemical Processes, Faculty of Materials Science and Technology, VSB-Technical University of Ostrava, 17. Listopadu 2172/15, Ostrava-Poruba, 708 00 Czech Republic; 2 Nanotechnology Centre, CEET, VSB-Technical University of Ostrava, 17. Listopadu 2172/15, Ostrava-Poruba, 708 00 Czech Republic; 3 Department of Physical Chemistry and Materials Science, University of Szeged, Rerrich Béla Tér. 1, Szeged, 6720 Hungary

**Keywords:** Graphitic carbon nitride, g-C_3_N_4_, Ag nanoparticles, Acid orange 7, Photocatalytic degradation, Materials for energy and catalysis, Chemistry, Engineering, Materials science

## Abstract

The main goal of this research is to investigate the effectiveness of graphitic carbon nitride (g-C_3_N_4_, g-CN) in both bulk and nanosheet forms, which have been surface-modified with silver nanoparticles (Ag NPs), as photocatalysts for the degradation of acid orange 7 (AO7), a model dye. The photodegradation of AO7 dye molecules in water was used to test the potential photocatalytic properties of these powder materials under two different lamps with wavelengths of 368 nm (UV light) and 420 nm (VIS light). To produce Ag NPs (Ag content 0.5, 1.5, and 3 wt%) on the g-CN materials, a new synthesis route based on a wet and low-temperature method was proposed, eliminating the need for reducing agents. The photodegradation activity of the samples increased with increasing silver content, with the best photocatalytic performances achieved for bulk g-CN samples and nanosheet silver-modified samples (with the highest content of 3 wt% Ag) under UV light, i.e., more than 75% and 78%, respectively. The VIS-induced photocatalytic activity of both examined series was higher than that of UV. The highest activities of 92% and 98% were achieved for the 1.5% Ag-modified g-CN bulk and nanosheet materials. This research presents an innovative, affordable, and environmentally friendly chemical approach to synthesizing photocatalysts that can be used for degrading organic pollutants in wastewater treatment.

## Introduction

Today, many of the technologies we commonly use have greatly benefited from advancements in nanoscale materials. These materials often exhibit unique or enhanced properties. Thus, future breakthroughs and significant advances in various vital technologies will largely depend on developing new materials at the nanoscale. To be competitive with commonly used materials, new materials must be cost-effective, environmentally friendly, and possess suitable properties. In addition, primary attention should be given to non-toxic materials as they have a much less negative impact on the environment and human health. Graphitic carbon nitride (g-C_3_N_4_, g-CN), a two-dimensional (2D) metal-free polymer material, is considered an excellent n-type semiconductor material due to its structural, optical, and physicochemical properties^1,2^. Due to its ease of preparation, low cost, and non-toxic properties, this substance has a wide range of applications in the fields of energy and environmental science^3–5^. For instance, it can be used for photocatalytic degradation of organic pollutants^6,7^, capturing and reducing carbon dioxide^8–11^, producing hydrogen^12,13^, and in solar cells^14,15^, fuel cells^16,17^, light-emitting devices^18,19^, sensors^20,21^, and many other fields^22–24^. In addition, it has a suitable medium band gap (2.7–2.8 eV) for efficient visible light harvesting, leading to the replacement or outperformance of commonly used materials such as titanium dioxide^25,26^. Unfortunately, g-CN suffers from a high recombination rate of the generated charge carriers, resulting in low photocatalytic efficiency^25,26^. Several strategies have been suggested to improve the photocatalytic activity of g-C_3_N_4_ under visible light. These include the formation of nanomaterials^27–29^ or porous structures^7,11,24^, doping^30,31^, deposition of metals or nonmetals^32–34^, and coupling of g-CN with other semiconductors^32,35,36^. The deposition of noble metals such as silver nanoparticles (Ag NPs) has gained much attention due to their enhanced electron storage capacity, lower cost, and non-toxicity^26,32,33^. The advantage of the extended exciton lifetime when pairing photocatalysts and Ag NPs is that silver forms a Schottky barrier that allows the capture of the photoexcited electron from the valence band of the semiconductor concerned^26,32,33^. The photodegradation activity increases with increasing lifetime of the electron–hole pairs^26,32,33^. Another phenomenon that favours the photodegradation activity associated with these composites in visible light is the surface plasmon resonance (SPR) effect^26,37,38^. Silver is a suitable candidate for surface modification of graphitic carbon nitride and has a more intense surface plasmon resonance effect and higher sensitivity to an appropriate radiation source than other noble metals^26,32,33,37,38^. In addition, it also improves the electron conductivity that can generate electron carriers and holes in photocatalysts^39,40^.

In the last decade, silver decorating has attracted the attention of many scientific groups. Much effort was devoted to the modification with silver nanoparticles, using melamine and silver nitrate (AgNO_3_) as precursors, for the photocatalytic degradation of methyl mercaptan^41^, methyl orange, p-nitrophenol^42^, sulfamethoxazole^43^, rhodamine B^44^ and methylene blue^45^. It is worth mentioning that silver-decorated g-C_3_N_4_ is also used for photocatalytic hydrogen evolution since Ag-modified g-C_3_N_4_ has strong photoinduced electron–hole separation capability compared to pristine g-C_3_N_4_^46–48^. Although silver nanoparticles are of great interest for decoration of graphitic carbon nitride, other silver compounds such as silver phosphate^49^, silver chromate^50^, silver carbonate^51^, silver oxide^52^, silver halides^53–56^ and others are used. The preparation of such modified g-C_3_N_4_ is more complex, and besides melamine and silver nitrate (AgNO_3_), disodium hydrogen phosphate (Na_2_HPO_4_), potassium chromate (K_2_CrO_4_), guanidine hydrochloride (CH_6_ClN_3_), sodium hydrogen carbonate (NaHCO_3_), cetyltrimethylammonium bromide (CTAB, C_19_H_42_BrN), sodium chloride (NaCl), sodium bromide (NaBr) and lithium iodide dihydrate (LiI × 2H_2_O) are also used. They are applied for purposes similar to those modified by silver nanoparticles, i.e., photocatalytic decomposition of organic compounds.

This study was conducted to investigate the effect of surface modification with Ag NPs on both bulk and nanosheet g-CN synthesized materials and to determine their potential use as photocatalysts in the degradation of acid orange (AO7) dye molecules. A low-temperature chemical approach was used to deposit Ag NPs on two types (bulk and nanosheet) of graphitic carbon nitride surface without using a reducing agent, making it a simple, straightforward, and cost-effective technique. The powders were analyzed using several physicochemical techniques, including X-ray powder diffraction (XRD), Raman spectroscopy, Fourier-transform infrared spectroscopy (FTIR), diffuse reflection spectroscopy (UV–Vis DRS), and photoluminescence (PL) spectroscopy, as well as scanning electron (SEM) and transmission electron (TEM) microscopy, and Mott-Schottky analysis. The study also investigated the photocatalytic properties of pristine and Ag NPs modified bulk and nanosheet g-CN powders during the photodegradation of the model dye acid orange 7 in ultraviolet (UV) and visible (VIS) light.

## Experimental

### Synthesis of g-C_3_N_4_-bulk and nanosheet materials and their surface modification with Ag nanoparticles

**Figure 1:**
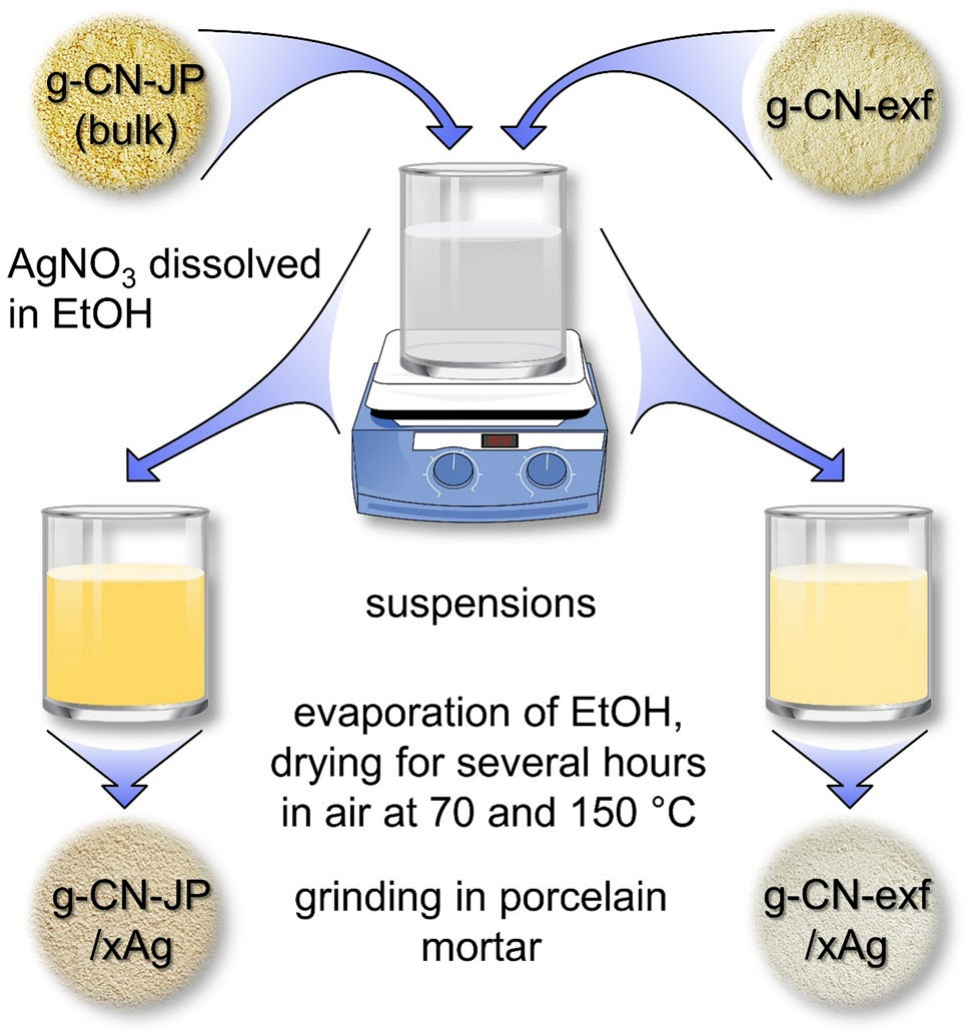
Flowchart of the preparation of silver-modified g-CN bulk and nanosheet powders.

The bulk g-C_3_N_4_ powder was prepared by heating melamine according to the work published elsewhere^36^. Melamine (3 g) was placed in a covered alumina crucible and calcined in a muffle furnace at 620 °C for 2 h and then at 550 °C for 1 h (heating rate: 15 °C/min) and allowed to cool freely in the furnace to room temperature. The sample was ground in a porcelain mortar to obtain a fine powder and labelled as g-CN-JP. Later, the as-prepared g-CN-JP powder (0.5 g) was placed in a covered alumina crucible and heated at 500 °C for 3 h (heating rate: 10 °C/min) in a muffle furnace, and the thermal exfoliation process was done. Upon the thermal exfoliation process of g-CN bulk (g-CN-JP), the resulting g-CN nanosheet material was received. The material was ground in a porcelain mortar to obtain a fine powder and labelled as g-CN-exf. Throughout the article, the terms g-CN bulk and g-CN-JP, as well as g-CN nanosheet and g-CN-exf, are used interchangeably. Both g-CN bulk and nanosheet materials were surface-modified with silver nanoparticles. The first stage involved dissolving pure silver nitrate (AgNO_3_, Lachema) in a 96% ethanol (EtOH) solution. The stoichiometric amount of silver nitrate used in the synthesis was 0.005, 0,015, and 0.03 g. In the second stage, 1 g of g-CN-JP or g-CN-exf powders were added to the as-prepared AgNO_3_-EtOH-H_2_O solutions. After forming a yellow homogeneously dispersed suspension, the mixtures were magnetically stirred for several hours. The yellowish suspensions were then air-dried for a few hours at 70 °C and then at 150 °C. The prepared samples were labelled as g-CN-JP-x% Ag and g-CN-exf-x% Ag, where x indicates the amount of Ag (0.5, 1.5, and 3.0 wt%) in the final composites. The composites were ground in a porcelain mortar to yield a fine powder. The samples labelled g-CN-JP-150 and g-CN-exf-150 represented materials obtained in the proposed low-temperature chemical synthesis based on their processing in ethanol solution only (without silver). The synthesis flowchart of silver-modified g-CN bulk and nanosheet powders is illustrated in Fig. 1.

### Instrumentation details

X-ray powder diffraction (XRD) analysis was employed to identify the crystalline phase of all pristine and silver-modified powders. The diffraction patterns were recorded using an Ultima IV diffractometer (RIGAKU, Japan). Working conditions of all studies were the following: CuK_α_ radiation (40 kV, 40 mA); K-beta filter; CBO selection slit—BB; Scintillation counter; continuous scan; Scan speed—2°/min; Step width—0.02°; Scan range—10–60° 2θ; Incident and receiving slit 1—2/3°; Receiving slit 2—0.6 mm.

The Fourier-transform infrared spectroscopy (FTIR) spectra were taken by a Nicolett™ Summit FT-IR spectrometer (Nicolet Instrument Company) with attenuated total reflection (ATR) detection mode using ZnSe-based ATR accessories. The spectra were collected in the range 4000–400 cm^−1^ with a resolution of 4 cm^−1^ and 16 scans. No additional sample preparation procedure was applied before the measurements.

Raman microscopic measurements were made using a Thermo Scientific, Waltham, MA, USA, DXR Raman microscope equipped with a 780 nm solid-state diode-pumped (DPSS) laser source. Exposure time was 1 s, and the number of exposures was 50 during data collection at each measurement. Gratings of 400 lines/mm and a 50 µm slit confocal aperture setting with a 10X objective was used. The spectral resolution was ~ 2 cm^−1^ in each case.

A Shimadzu Ltd. UV-2600 Series spectrometer with a 60 mm diameter Shimadzu Ltd. IRS-2600Plus integrating sphere was employed to evaluate the UV–Vis DRS spectra of dry powder samples at room temperature between 220 and 800 nm. A reference sample of powder BaSO_4_ was utilized. The reflectance data were transformed using the Kubelka-Munk (K.-M.) function, and the indirect bandgap energies (E_g_) values were determined using Tauc's plot.

The photoluminescence (PL) emission spectra of all derived g-C_3_N_4_-based compounds were assessed at room temperature using an Edinburgh Instrument Ltd FLSP920 Series spectrometer in the wavelength range of 350–600 nm. The spectrometer used a 450 W non-ozone xenon lamp (Steady state Xe900 lamp) and an R928P detector (PMT detector). In this case, the Czerny-Turner configuration was used. The absorption spectra were measured using an Edinburgh Instruments, Ltd. FLSP920 Series spectrometer.

The morphology of the powder samples was investigated using a JEOL JSM-7610F Plus scanning electron microscope (SEM) (Tokyo, Japan) with a Schottky cathode at 15 keV in a high vacuum chamber. Powder samples were prepared on brass stubs with carbon tape and coated with a 13 nm nanometric layer of gold. Elemental composition analysis and mapping were performed using the energy-dispersive X-ray spectroscope (EDS) AZtec Ultima Max 65 (Oxford Instruments, Abingdon, UK).

The TEM images were recorded in a Jeol JEM-1400plus type microscope (Japan) at 120 keV. Prior to measurement, one drop of the ethanol-dispersed sample was introduced onto a Formvar foil film-covered copper grid and left to dry. The JMicroVision software was used to determine the average particle diameter of the silver NPs on the g-CN material.

### Electrochemical investigations

10 mg of each sample was dispersed in 5 ml of deionized water and then ultrasonically homogenized for 30 min. Of this suspension, 30 μl was dropped onto the surface of a glassy carbon electrode, and then this assembly was dried at 85 °C for 3 h. Electrochemical tests were performed on a Metrohm Autolab PGSTAT302 potentiometer, with a glassy carbon electrode (GCE), Ag/AgCl (3M KCl), and a Pt sheet serving as the working, reference, and counter electrode. Before taking each measurement, the electrode surface was cleaned with a polishing set for solid-state electrodes. An aqueous solution of 0.1 M KCl was used as an electrolyte, which was bubbled with nitrogen for 30 min before electrochemical measurement. The Mott-Schottky recordings were taken at an AC frequency of 300 Hz with an amplitude of 10 mV.

### Photocatalytic activity measurements

The photocatalytic activity of all g-C_3_N_4_-based materials was determined by degrading acid orange 7 (AO7) dye. Under UV (368 nm) and VIS (420 nm) light, photodegradation of an aqueous solution of AO7 at a concentration of 7.14 × 10^−4^ mol/l was conducted. In a typical procedure, 50 mg of catalyst was dispersed in a glass beaker in deionized water and magnetically stirred at 300 rpm at room temperature to obtain a homogeneously dispersed suspension. After that, AO7 was added to the suspension. All as-prepared suspensions were magnetically stirred at 300 rpm for 1 h at room temperature in dark conditions to estimate the adsorption and desorption equilibrium of AO7 dye on the catalyst. Afterwards, a UV lamp of wavelength 368 nm or VIS lamp of wavelength 420 nm was switched on for ultraviolet or visible light irradiation measurements. During the specified experimental time, two millilitres of a uniformly dispersed suspension of the test material in demineralized water with AO7 dye were collected using a syringe. The collection was done in the dark or under UV/VIS light. Afterwards, the suspensions were filtered using a syringe filter with a pore size of 0.45 µm (CHROMAFIL GF/RC-20/25 filters, Macherey–Nagel, Germany). The absorbances of the filtered suspensions were measured at 480 nm to separate the photocatalytic material. The degradation of AO7 was monitored in a 1 cm diameter quartz glass microcuvette in the presence of demineralized water using a Helios Epsilon spectrometer set at 480 nm. For all studied samples, the percentage photocatalytic activity degradation of AO7 (PA_sample_) was calculated according to the equation: PA_sample_ = (1 − (C_eq. AO7_/C_0 AO7_)) × 100%, where C_eq. AO7_ is the equilibrium state of AO7 concentration, and C_0 AO7_ is AO7’s initial concentration.

## Results and discussion

### XRD studies

**Figure 2:**
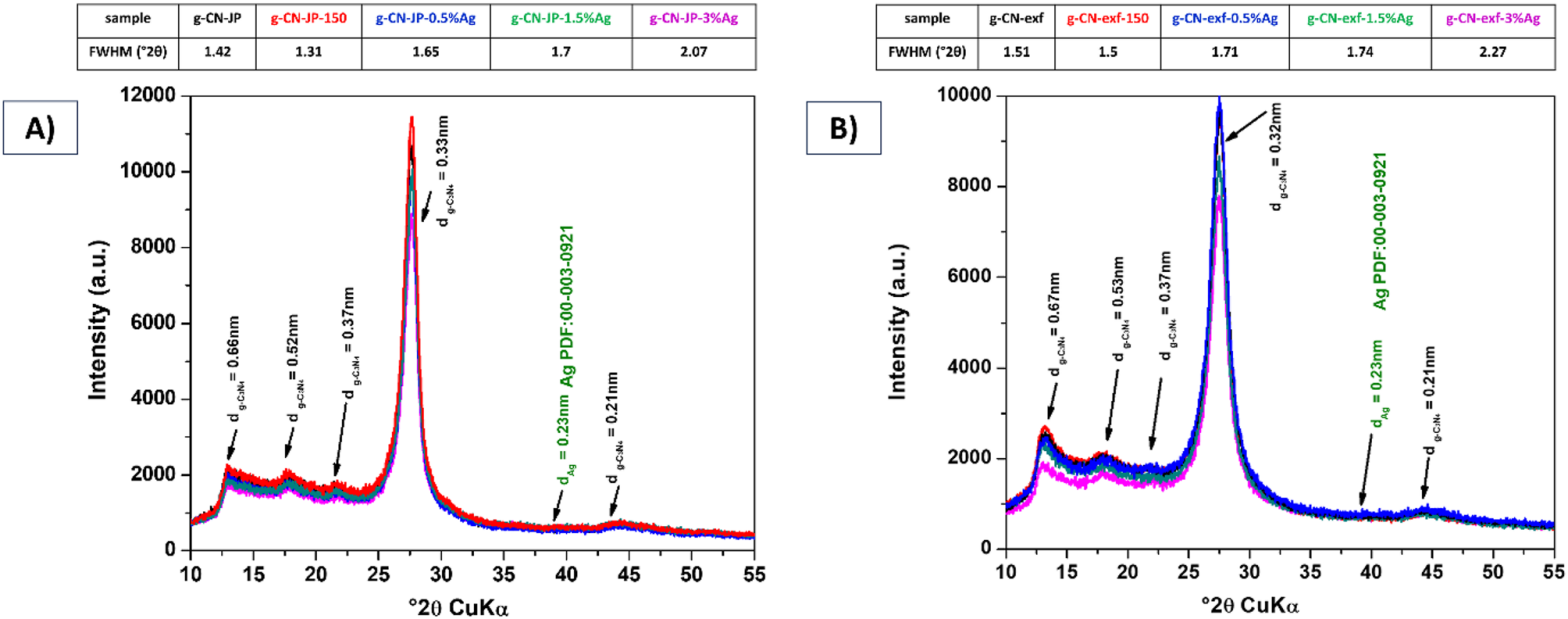
XRD patterns of silver-modified g-CN bulk (**A**), and nanosheet (**B**) materials.

The measured samples represent semi-crystalline materials (Fig. 2A,B). The XRD pattern of silver-modified g-CN bulk materials (Fig. 2A) shows five diffraction lines at °2θ CuK_α_ with the interlayer distance of the planes calculated according to Bragg´s equation: 12.7 (d = 0.66 nm), 17.7 (d = 0.52 nm), 21.9 (d = 0.37 nm), 27.5 (d = 0.33 nm) and 44° (d = 0.21 nm) which can be assigned to the hexagonal phase of g-C_3_N_4_ (ICDD:87–1526)^57–59^. The corresponding diffraction lines (*hkl* positions) are (100), (110), (111), (002), and (103)^57–59^. The strongest diffraction line at 2θ = 27.5° is associated with the interlayer stacking of the (002) g-C_3_N_4_ planes. The diffraction line at 2θ = 12.7° is attributed to the (100) planes that correspond to the in-plane ordering of the nitrogen-linked heptazine units^59–61^. Again, the hexagonal phase of g-C_3_N_4_ (ICDD:87-1526)^57–59^ can be assigned to the five diffraction lines at °2θ CuK_α_ that are visible in the XRD pattern of silver-modified g-CN nanosheet series of samples (Fig. 2B). The interlayer distance of the planes was calculated using Bragg´s equation, and the values are 12.7 (d = 0.67 nm), 17.7 (d = 0.53 nm), 21.9 (d = 0.37 nm), 27.5 (d = 0.32 nm), and 44° (d = 0.21 nm).

The silver-modified g-CN nanosheet (thermally exfoliated) samples (Fig. 2B), in contrast to the bulk samples, are visibly less ordered (Fig. 2A), as confirmed by full width at half maximum (FWHM) evaluation of the most intensive peak d = 0.3 nm of the g-C_3_N_4_ phase. The width of nanosheet (thermally exfoliated) samples (Fig. 2B) is more significant than that of non-exfoliated samples (Fig. 2A). As the amount of silver content increases, there is a corresponding increase in the width observed. This is another phenomenon that has been noted. The most disordered material was the nanosheet (thermally exfoliated) sample with 3 wt% of silver (g-CN-exf-3%Ag). It should be noted that the peak of the silver phase is difficult to detect as its amount and crystalline state are very low.

### Spectroscopy (FTIR, Raman, DRS, PL) studies

**Figure 3:**
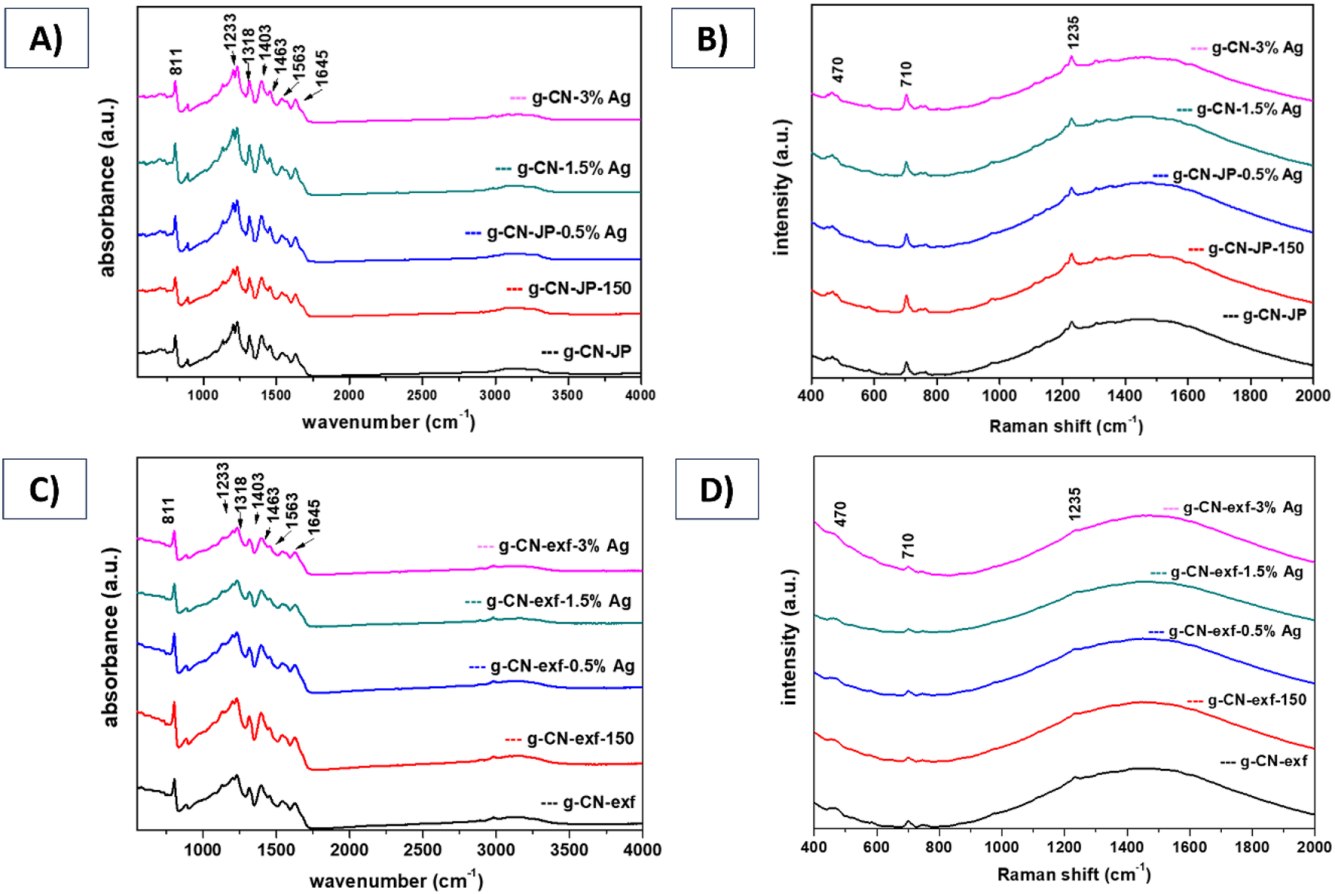
FTIR (**A, C**) and Raman spectra (**B, D**) of silver-modified g-CN bulk, and nanosheet materials.

The IR spectra of both analyzed sample series are shown in Fig. 3A,C. The recordings show characteristic sharp absorption peaks at 811, 1233, 1318, 1403, 1463, 1563, and 1645 cm^−1^ and broad absorption peaks in the region 3000–3700 cm^−1^ due to N–H hydrogen bonds and -OH bonds.

The sharp band at 811 cm^−1^ was assigned to the breathing mode of out-of-plane vibrations of triazine units. The very strong absorption band at 1233 cm^−1^ and strong band at 1318 cm^−1^, 1403 cm^−1^ correspond to the C–N stretching vibrations (in which the C–NH–C and C–C vibrations are probably involved). The band at 1463 cm^−1^ corresponds to the number of rings of the heterocyclic structure, and the strong bands at 1563 cm^−1^ and 1645 cm^−1^ indicate stretching vibrations of the C=N bonds of g-C_3_N_4_ heterocycles^62,63^. The wide absorption peaks observed between 3000 – 3700 cm^−1^ consist of several components. Among them, bands at 3100 cm^−1^ and 3300 cm^−1^ can be attributed to hydrogen bonds, while those at 3350 cm^−1^ can be assigned to -OH water groups. The remaining components of this broad band can be assigned to the N–H stretching of the remaining amino groups^64^. The analyzed IR spectra were similar for silver-modified g-CN bulk and nanosheet series. This may be due to the detection limit of the method, with no additional bands corresponding to Ag content occurring.

The Raman spectra of all investigated samples are presented in Fig. 3B,D. Three characteristic peaks of graphitic carbon nitride at 470 cm^−1^, 710 cm^−1^, and 1235 cm^−1^ can be recognized. In addition, Raman shift is recorded in the measured spectrum range 400–2000 cm^−1^. These bands are assigned to the N–C–N stretching vibrations, bending vibrations = C (sp^2^), and the vibrations of C–N bonds in the heterocycle of g-C_3_N_4_^65^, as confirmed by FTIR studies. It is worth noting that there were no observable changes in the Raman spectra after the silver modification of both bulk and nanosheet g-CN materials. However, the impact of the exfoliation process from bulk to nanosheet g-CN material is evident as the intensity of the Raman spectral bands in the non-exfoliated series showed a significant decrease compared to the exfoliated series (as shown in Fig. 3B,D).

**Figure 4:**
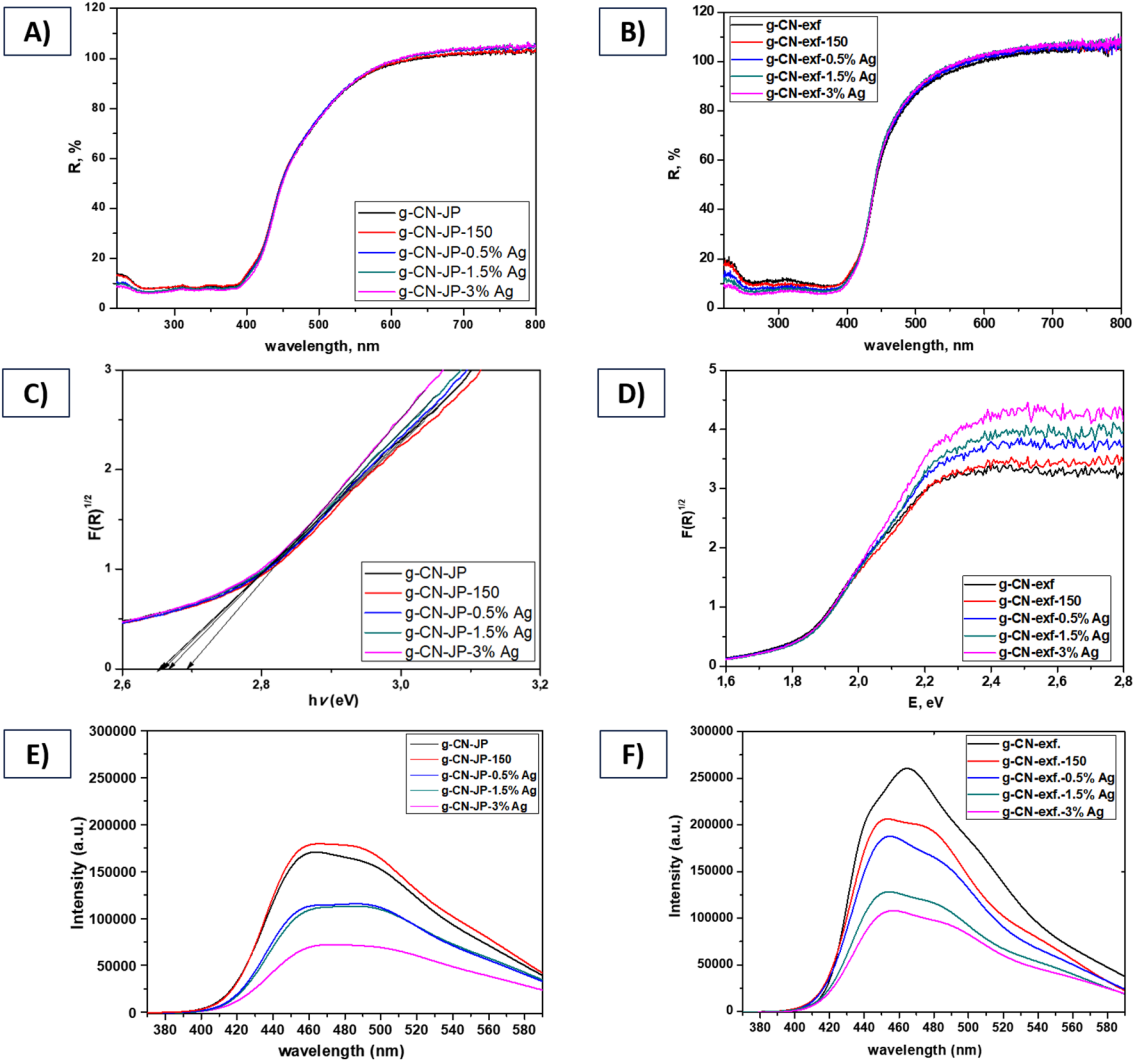
UV–Vis DRS (**A, B**) and Tauc spectra (**C, D**) at wavelengths from 220 to 800 nm and photoluminescence spectra (**E, F**) of silver-modified g-CN bulk (**A, C, E**), and nanosheet (**B, D, F**) materials.

The optical properties of the silver-modified g-CN bulk and nanosheet materials were evaluated using diffuse reflectance spectroscopy (Fig. 4A–D). The Tauc method was used to generate a Tauc plot by analyzing the reflectance spectra of all samples (as shown in Fig. 4C,D). This method involves transforming the UV–Vis reflectance spectrum into a modified Kubelka–Munk function, plotting it against photon energy (i.e., hv, where h represents the Planck constant and v represents the photon's frequency), and determining the E_g_ value by extrapolating the slope to zero.

The band gap energies (E_g_) for silver-modified g-CN bulk and nanosheet materials were 2.66–2.69 eV and ca. 1.70 eV, respectively. The results of the calculated E_g_ are presented in Table 1. It should be noted that the silver content ranged only from 0.5 to 3 wt%. Therefore, the effect of increasing Ag concentration on the surface of g-CN in both series of materials, and thus the obtained results in E_g_ values, is negligible. It can be observed that both g-CN-JP-x%Ag and g-CN-exf-x%Ag series show an increase in E_g_ value with an increase in Ag content. The samples belonging to the g-CN-exf-x%Ag and g-CN-JP-x%Ag series, which have been modified with Ag, exhibit a notable rise in absorption in the visible light range spanning from 400 to 800 nm (see Fig. 4A,B). This growth in absorption is due to the Surface Plasmon Resonance (SPR) phenomenon caused by the Ag nanoparticle effect^26,37,66,67^. The emission peak observed at 472 nm for g-CN-JP and g-CN-JP-150 can be attributed to electron transitions between antibonding π* and bonding π states and between π* and lone pairs of electron states^68,69^. As mentioned in this study^70^, nitrogen loss of C_3_N_4_ was observed because of its annealing.

**Table 1:** Indirect band gap energy (E_g_) values (Kubelka–Munk function, Tauc spectra) from DRS, maximum emission bands from photoluminescence spectra of silver-modified g-CN bulk and nanosheet materials.

Materials	E_g_ (eV)	λ_max_ (nm)
g-CN-JP	2.66	472
g-CN-JP-150	2.65	473
g-CN-JP-0.5%Ag	2.66	476
g-CN-JP-1.5%Ag	2.67	478
g-CN-JP-3%Ag	2.69	479
g-CN-exf	1.79	464
g-CN-exf-150	1.80	469
g-CN-exf-0.5%Ag	1.79	464
g-CN-exf-1.5%Ag	1.80	462
g-CN-exf-3%Ag	1.80	468

Furthermore, the optical properties were measured using photoluminescence (PL) spectra analysis at room temperature. The results are depicted in Fig. 4E,F. Both series of studied materials showed decreasing PL intensity with increasing Ag content. The silver-modified g-CN bulk and nanosheet series exhibit a red shift in the PL maxima (λ_max_) from 472 to 479 nm and 464 to 468 nm, respectively. Additionally, this shift in emission peaks is accompanied by a decrease in the PL emission bands, as presented in Table 1^2,37,71^.

The absorption spectra of the silver-modified g-CN bulk and nanosheet materials were measured, and the estimated results are included in the Supplementary Material in Fig. S1A,B. The absorption spectrum of g-CN-JP (Fig. S1A) displays an absorption edge at approximately 470 nm, which corresponds well with the band gap energy (E_g_ = 2.66 eV, as shown in Table 1), as reported by Cao et al.^72^ and Cao and Yu^13^. The absorption edges for other composites (g-CN-JP-x% Ag) shown in Fig. S1A are located within the bandgap energy range of about 470–480 nm. Fig. S1B shows the visible absorption edges of pristine materials g-CN-exf, g-CN-exf-150, and their composites (g-CN-exf-x% Ag) in the range of 463–470 nm^13,72^. The optical band absorption edges are depicted for g-CN-exf, g-CN-exf-150, and their composites (g-CN-exf-x% Ag), and they are shifted towards higher wavelengths. This shift is causing a decrease in the band gap energies compared to pristine g-CN-JP, g-CN-JP-150, and their composites (g-CN-JP-x% Ag), respectively. The increase in the bond length of the sp^2^ C-N clusters during the thermal exfoliation process may be due to the extension of the polymeric network of g-C_3_N_4_ by more linking tris-triazine units, as illustrated in Fig. S1A,B. This phenomenon is a typical feature of thermal exfoliation^73^ and agrees with the band gap energies (see Table 1).

### Morphology (SEM, TEM) examination

**Figure 5:**
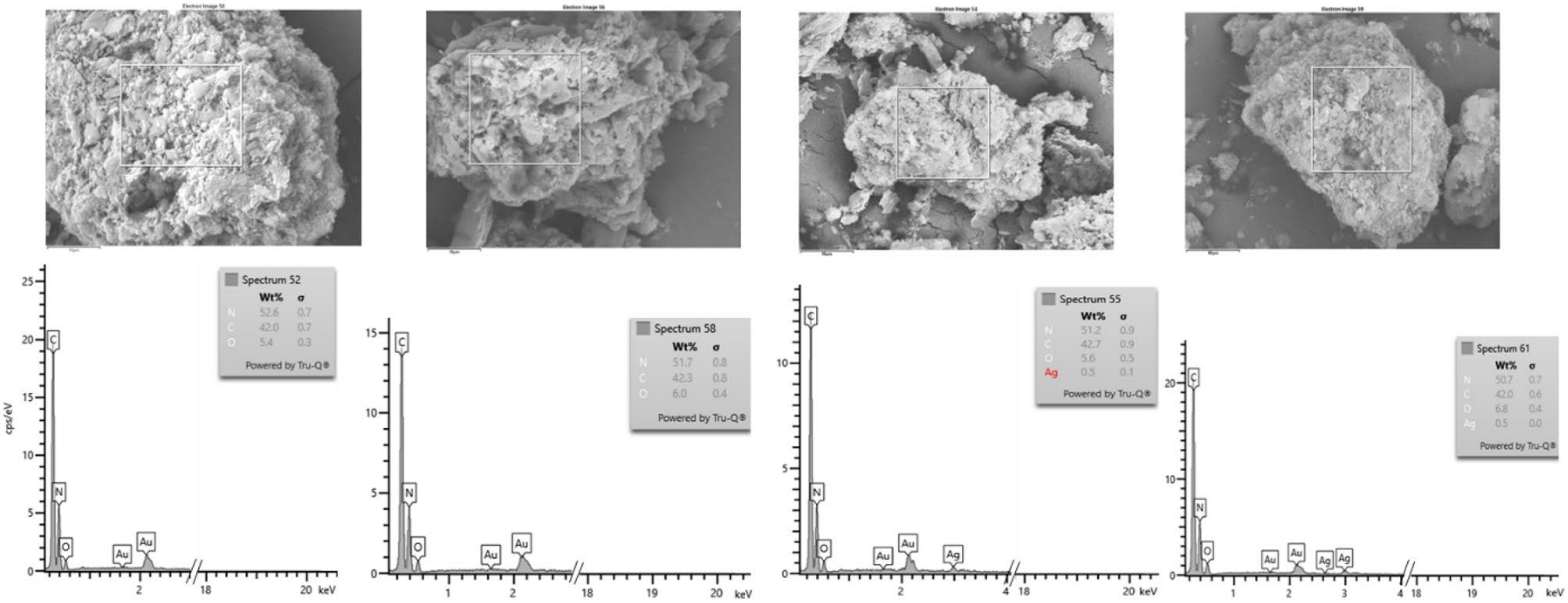
SEM images of silver-modified g-CN bulk and nanosheet materials (from left to right g-CN-JP, g-CN-exf, g-CN-JP-3%Ag, g-CN-exf-3%Ag).

The energy dispersion microanalysis (EDS) method was used to confirm the presence of silver in samples with the highest content (3 wt%), see Fig. 5. Samples were coated with gold (10 nm thick layer) to avoid charging during observation in an electron microscope chamber. The measured values for both samples g-CN bulk and nanosheet—are basically the same and around the detection limit—0.3–0.5% wt. For samples containing 0.5 and 1.5 wt% of silver, this method may not detect it. It should also be noted that the quantification of light elements such as C, N, and O is biased and has a higher standard deviation when using EDS microanalysis. However, this method is suitable for confirming the presence of Ag in the sample with its highest content and complements the results of other analyses. Additionally, the SEM images (secondary electrons regime) of all silver-modified g-CN bulk and nanosheet materials at two magnifications: 2500× (scale bar 10 µm) and 17000x (scale bar 1 µm) are provided in Supplementary Material in Fig. S2.

**Figure 6:**
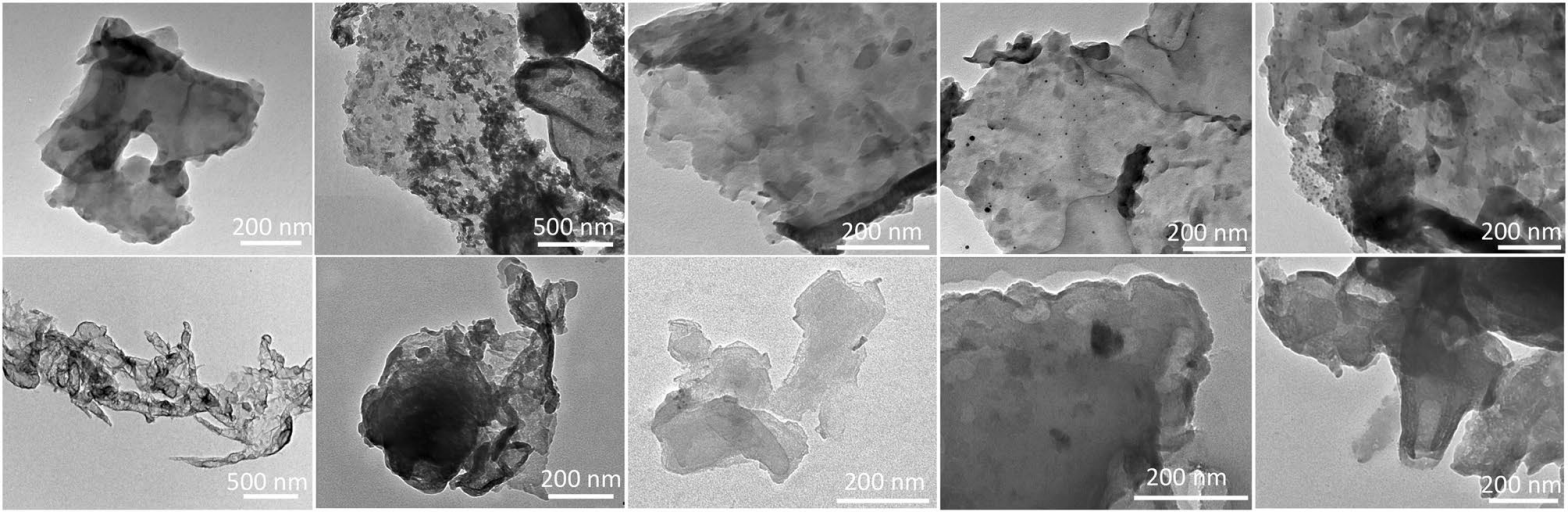
TEM images of silver-modified g-CN bulk and nanosheet materials (from left to right g-CN-JP, g-CN-JP-150, g-CN-JP-0.5%Ag, g-CN-JP-1.5%Ag, g-CN-JP-3%Ag).

The TEM images of the samples indicate a similar structure to that presented in the SEM images. Contrary to the EDS’s detection limit, silver nanoparticles can be detected on the g-CN-JP materials (Fig. 6), and their increasing amount can also be observed as the Ag content increases.

**Figure 7:**
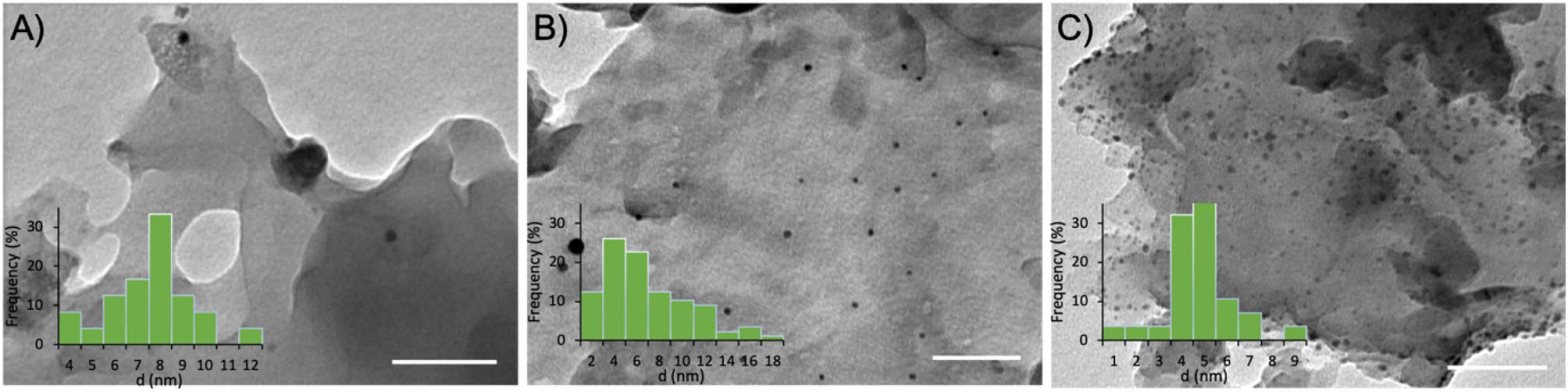
TEM images of silver-modified g-CN bulk materials, g-CN-JP-0.5%Ag (**A**), g-CN-JP-1.5%Ag (**B**), and g-CN-JP-3%Ag (**C**), the scale bars represent 100 nm. The insets represent particle size distribution functions of each sample.

For verification, the following figure (Fig. 7) clearly shows the presence of Ag nanoparticles as the Ag content increases. The particle size distribution was also analyzed, and a decrease in the average diameter for the Ag nanoparticles, from 8.1 ± 1.9 nm to 5.2 ± 1.6 nm, was observed as the Ag content varied from 0.5 to 3 wt%, respectively.

The presence of Ag is proved based on the EDS results; however, they are not clearly/unquestionably distinguished on the “exfoliated” nanosheets except for the sample with 3% Ag content, indicating further differences between the systems. One possible explanation is that the NPs are smaller in the nanosheets.

### Electrochemical measurements

To further investigate the efficiency of the photocatalyst, Mott-Schottky measurements were performed. This technique relies on applying a bias voltage to modulate the width of the space-charge interfacial double layer and, therefore, the value of capacitance when the solution side of the interface can be approximated by the Helmholtz model of the double layer^74^. The Mott-Schottky Eq. (1) describes the relation between the space charge capacitance C and the applied potential V.

1 $$\frac{1}{{{\text{C}}^{2} }} = \frac{2}{{\upvarepsilon_{{\text{r}}} \upvarepsilon_{0} {\text{eN}}_{{\text{D}}} }}\left( {{\text{V}} - {\text{V}}_{{{\text{FB}}}} - \frac{{{\text{kT}}}}{{\text{e}}}} \right)$$

where ε_r_ is the dielectric constant, ε_o_ is the dielectric constant of vacuum, N_D_ is the dopant density for n-type semiconductor, T is the temperature, K is the Boltzmann constant and e is the elementary electron charge. This equation is applicable in the linear part of the Mott–Schottky plot.

**Figure 8:**
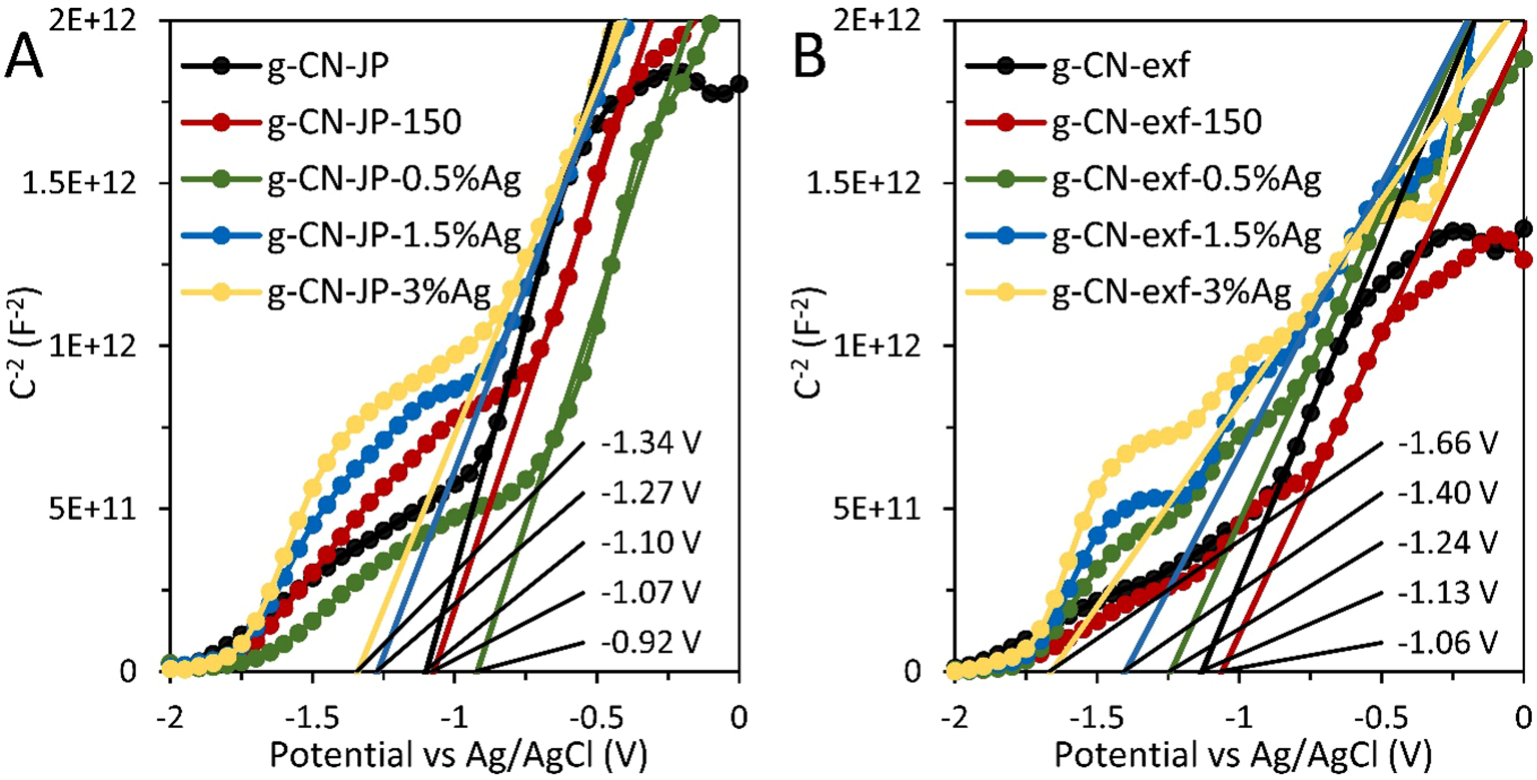
Mott-Schottky plots at 300 Hz for silver-modified g-CN bulk (**A**) and nanosheet (**B**) materials. The flat band potential values are shown in the plots.

The values of flat band potentials (V_FB_) were calculated as the intercepts of the extrapolation lines with the potential axis in the Mott–Schottky plot, which is the function of the inverse of the measured capacitance squared on the potential (Fig. 8).

For n-type semiconductors, it is assumed that flat band potential is nearly identical to the conduction band (V_CB_) edge potential according to Eq. (2)^75,76^.

2 $${\text{V}}_{{{\text{CB}}}} \approx {\text{V}}_{{{\text{FB}}\left( {{\text{NHE}}} \right)}} = {\text{V}}_{{{\text{FB}}}} + \Delta {\text{V}} - 0.059 \cdot (7 - {\text{pH}}_{{0.1{\text{MKCl}}}} )$$

where V_FB(NHE)_ is the flat band potential recalculated against NHE electrode at pH = 7, ∆V is the potential of Ag/AgCl reference electrode vs NHE.

The valence band edge potential (V_VB_) was calculated using Eq. (3)^75,76^.

3 $${\text{V}}_{{{\text{VB}}}} = {\text{V}}_{{{\text{CB}}}} + \frac{{{\text{E}}_{{\text{g}}} }}{{\text{e}}}$$

where E_g_ is the band gap energy.

When the light interacts with the photocatalysts, holes are formed in the valence band, whereas electrons are transferred to the conduction band. The reactions concerning the formation of $\cdot {\text{O}}_{2}^{ - }$ and $\cdot {\text{OH}}$ can be ascribed as follows (Eqs. 4–6).

4 $${\text{OH}}^{ - } + {\text{h}}^{ + } \to \cdot {\text{OH}}$$

5 $${\text{O}}_{2} + {\text{e}}^{ - } \to \cdot {\text{O}}_{2}^{ - }$$

6 $${\text{H}}^{ + } + \cdot {\text{O}}_{2}^{ - } \to \cdot {\text{HO}}_{2}$$

**Figure 9:**
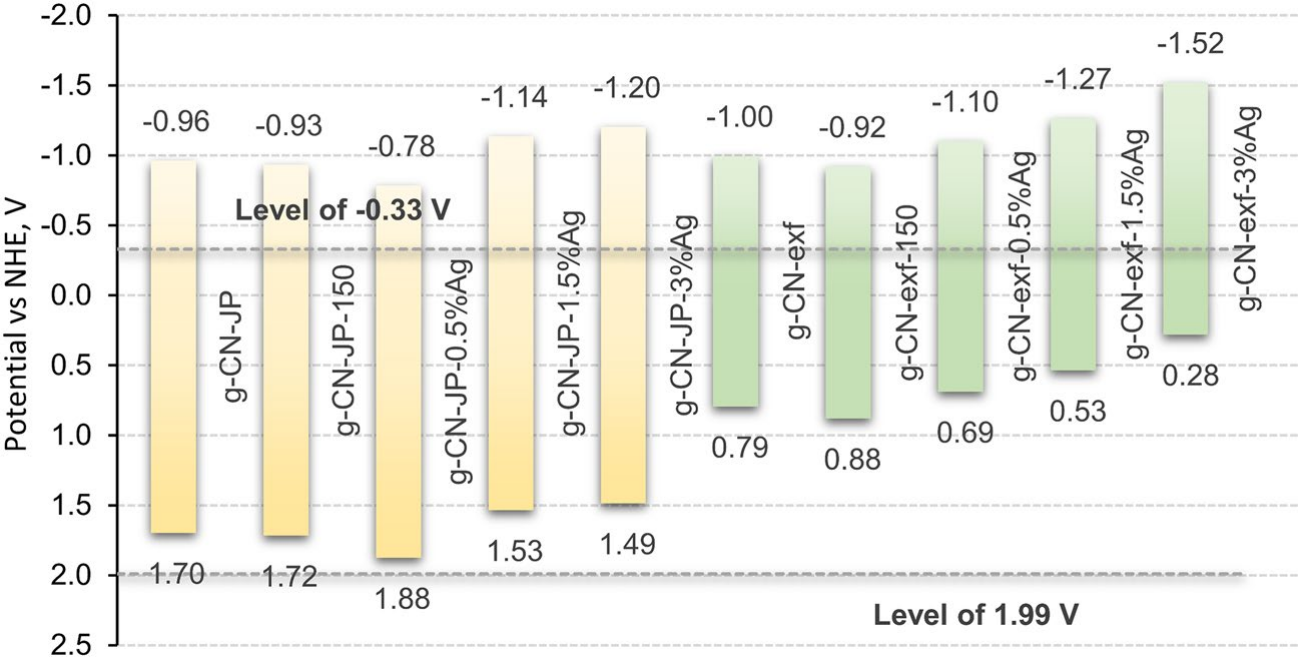
Energy diagrams of CB, VB edge potentials and energy gaps for silver-modified g-CN bulk (yellow) and nanosheet (green) samples.

The CB, VB edge potentials and band gap energies are given in Fig. 9. For all samples, the energy value of the CB edge potential is higher than − 0.33 V, indicating that only superoxide radicals are formed after irradiation. Since the position of the valence band edge potential is much lower than the standard redox potential $\cdot {\text{OH}}^{ - } / \cdot {\text{OH}}$, the formation of hydroxyl radicals is improbable. The shift of V_CB_ to more negative values and deviating from the standard redox potential of $\cdot {\text{O}}_{2} / \cdot {\text{O}}_{2}$ pair indicates an increase in the ability of the conduction band electrons of the given samples to form superoxide radicals ($\cdot {\text{O}}_{2}^{ - }$), and this ability was highest for the samples containing 1.5 and 3 wt% Ag, both in the bulk and nanosheet series.

### Degradation of AO7 under UV or VIS light

**Figure 10:**
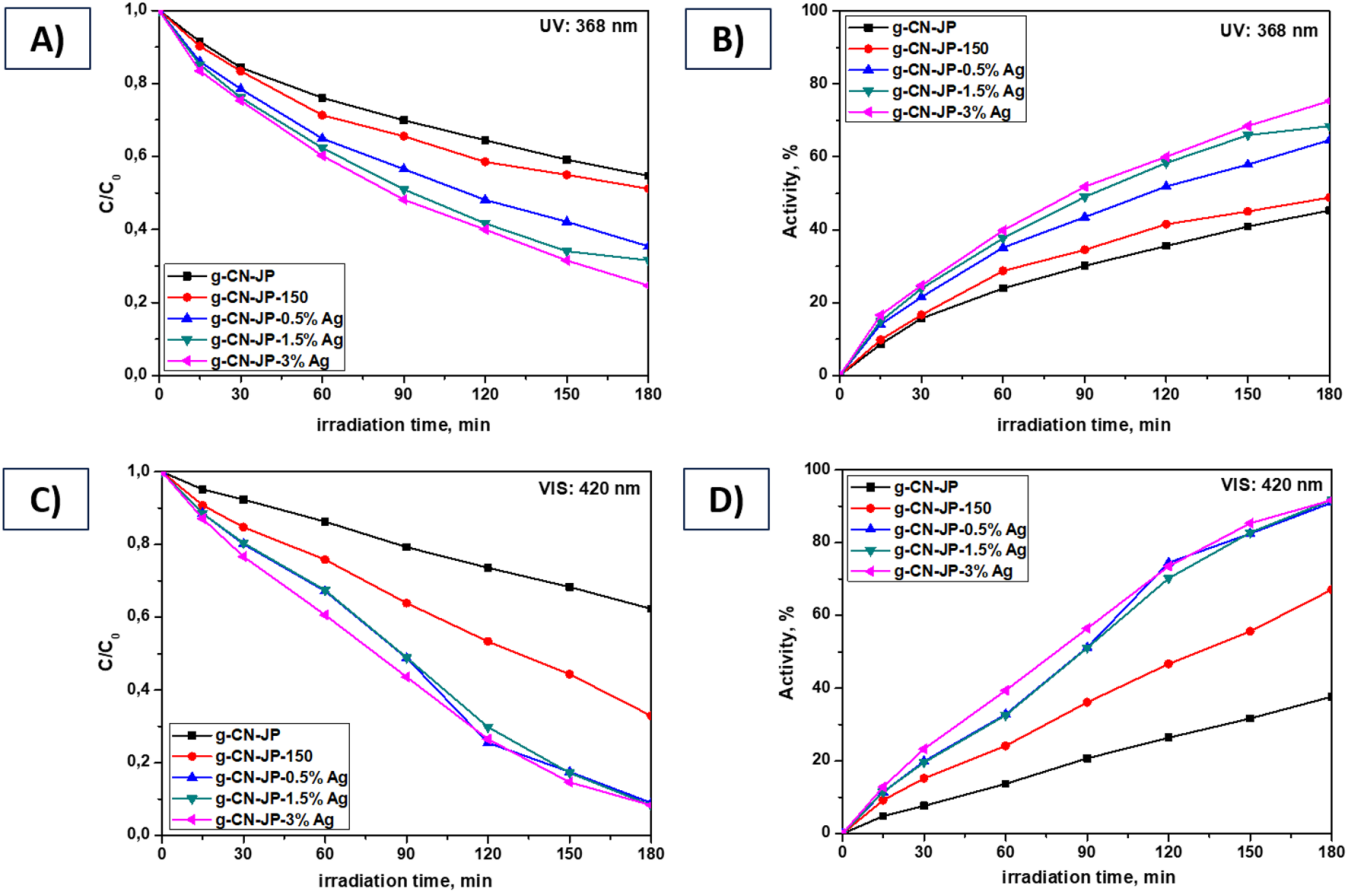
Photocatalytic degradation C/C_0_ and activity processes of silver-modified g-CN bulk materials. Comparison of the UV lamp: 368 nm (**A, B**) vs. VIS lamp: 420 nm (**C, D**).

**Figure 11:**
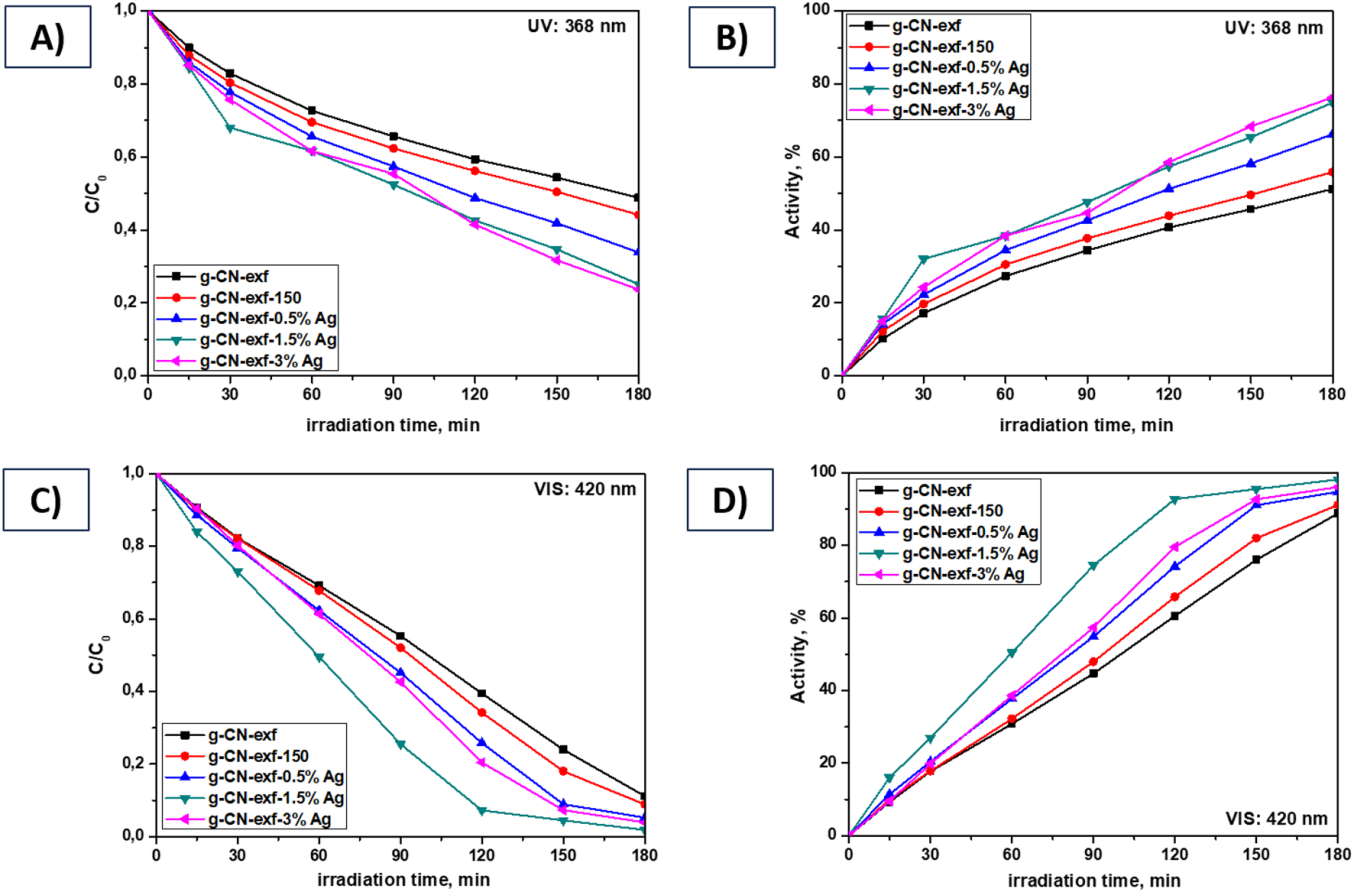
Photocatalytic degradation C/C_0_ and activity processes of silver-modified g-CN nanosheet materials. Comparison of the UV lamp: 368 nm (**A, B**) vs. VIS lamp: 420 nm (**C, D**).

The photocatalytic degradation of the AO7 using silver-modified g-CN bulk and nanosheet photocatalysts was examined under UV (368 nm) and VIS (420 nm) light. The results are presented in Figs. 10 and 11 and Table 2. Further, during all photodegradation processes using UV/VIS lamps, the photolysis process of the AO7 dye was also performed, and the results are shown in Supplementary Material in Fig. S3. These findings demonstrated that AO7's stability under UV/VIS light irradiation did not cause a significant change in its absorbance (see Fig. S2). Figure 10A–D shows that the photocatalytic performance of the pristine g-CN bulk material (sample g-CN-JP) was limited compared to the g-CN bulk material prepared in EtOH-H_2_O solution and heat-treated at 150°C in the air (sample g-CN-JP-150). The most noticeable difference between the g-CN-JP (38%) and g-CN-JP-150 (67%) samples is observed during the 3 h of the photodegradation process of AO7 under visible light (refer to Fig. 10C,D). The photocatalytic properties are enhanced by a small amount of Ag (only 0.5 wt%), observed within 15 min after turning on the UV or VIS lamp. The effect of silver-modified samples within only 0.5 wt% was observed in our previous work when the composite melem/g-C_3_N_4_ served as a main photocatalytic material^26^. The photocatalytic activities of analyzed samples under UV and VIS lamps increase due to the increasing Ag content. This indicates that the added Ag NPs had a considerable influence on the photocatalytic behavior of g-CN bulk material, and the surface modification succeeded in obtaining g-CN-JP-x% Ag composite photocatalysts. For the silver-modified g-CN bulk materials, the photodegradation activities under UV and VIS light were between 45–75% and 38–92%, respectively. The highest photodegradation activities (after 3 h of irradiation) were achieved for the sample with the 1.5 and 3 wt% Ag. A study by Alenazi and co-authors^77,78^ presented a similar observation for the surface modification of g-C_3_N_4_ material with Mo^6+^ and W^6+^ in the composites Mo^6+^@g–C_3_N_4_ and W^6+^@g-C_3_N_4_, respectively, at concentrations between 0.5 and 3 wt%. The Mo^6+^@g–C_3_N_4_ composites were tested to remove the potential pollutant 2,4-dichlorophenoxyacetic acid under natural sunlight^77^. The W^6+^@g-C_3_N_4_ composites were tested for removing chlorophenol derivatives in natural sunlight exposure^78^. The works mentioned clearly indicate that modifying g-C_3_N_4_ in an amount of 0.5–3 wt% is sufficient to achieve the desired effects, making the process cost-effective. It can also be argued that the photocatalytic activity of bulk g-CN surface-modified with Ag nanoparticles is satisfactory under visible light due to suppressing the recombination of photogenerated electron/hole pairs. Considering the g-CN nanosheet materials (g-CN-exf, g-CN-exf-150) (Fig. 11), it is evident that the photodegradation activities achieved under UV/VIS light are higher than those of the pristine g-CN bulk samples (g-CN-JP, g-CN-JP-150) (Fig. 10). These results generally indicate the efficiency of the thermal exfoliation process carried out for the g-CN bulk material. After 3 h of UV to VIS irradiation, the difference in photocatalytic activity for unmodified g-CN nanosheet materials over AO7 was 35%. The thermal exfoliation process increases dye molecules' adsorption capacity, leading to a better ability to suppress the recombination of charge carriers^73,79–81^. Under UV and VIS light, the photodegradation activities for the nanosheet silver-modified g-CN materials ranged from 51 to 78 and 89–98%. The VIS-induced photocatalytic activity of both examined series was higher than that of UV. It is also clearly visible that the highest activities were achieved for the silver-modified g-CN nanosheet material. The Ag NPs that were used to modify the surface of g-CN bulk and nanosheet materials acted as photoinduced electron collectors, allowing them to be segregated from holes, as demonstrated in Fig. 4, which resulted in a decrease in PL intensity as well as an increase in the photocatalytic degradation processes towards AO7 dye. The silver-modified g-CN nanosheet material showed the highest activity (Fig. 11, Table 2). Supplementary Material includes a literature survey of composites of graphitic carbon nitride (g-C_3_N_4_, g-CN) with silver nanoparticles that were utilized for photodegradation processes involving selected dyes, such as methyl orange (MO), methylene blue (MB), rhodamine B (RhB), and acid orange 7 (AO7). This survey is presented in Table S1. The presented results demonstrate the effectiveness of utilizing metallic silver nanoparticles to modify the surface of a graphitic carbon nitride material. Our work focuses on the cost-effective and straightforward approach that we employed to modify the surface of g-CN bulk and nanosheet materials containing silver nanoparticles at concentrations of 0.5, 1.5, and 3 wt% without the use of a reducing agent. The resulting materials were then investigated as photocatalysts in wastewater treatment towards acid orange 7 dye under UV and VIS irradiation.

**Table 2:** Activity and kinetic constant (after 3 h) of silver-modified g-CN bulk and nanosheet materials. Comparison of the UV (360 nm) vs. VIS (420) nm lamps.

Materials	UV lamp		VIS lamp	
	Activity (%)	k_cal_ 10^−3^ (min^−1^)	Activity (%)	k_cal_ 10^−3^ (min^−1^)
g-CN-JP	45	4.4	38	2.7
g-CN-JP-150	49	5.1	67	5.5
g-CN-JP-0.5%Ag	65	7.0	91	9.5
g-CN-JP-1.5%Ag	68	8.0	92	9.4
g-CN-JP-3%Ag	75	8.8	92	10.5
g-CN-exf	51	5.1	89	7.9
g-CN-exf-150	56	5.9	91	8.7
g-CN-exf-0.5%Ag	66	7.1	95	10.9
g-CN-exf-1.5%Ag	75	8.7	98	16.2
g-CN-exf-3%Ag	78	8.2	96	11.5

The difference in degradation rates between the silver-modified g-CN bulk and nanosheet series under UV and VIS light is also depicted in Figs. 10 and 11, and this finding is further supported by estimated kinetic constants (Table 2). The pseudo-first-order reaction equation^26,77,78^ ln(C/C_0_) = k_cal_t, where C_0_ is the initial concentration of AO7, C is the concentration of AO7 at the time t of the measurement, and k is the kinetic constant, was used to derive the calculated kinetic constants (k_cal_t) for all materials. The obtained kinetic constants for silver-modified g-CN bulk materials were 4.4–8.8 × 10^−3^ min^−1^ (under UV lamp) and 2.7–10.5 × 10^−3^ min^−1^ (under VIS lamp). For the silver-modified g-CN nanosheet materials series, the determined kinetic constants under UV and VIS light were in the ranges 5.1–8.7 × 10^−3^ min^−1^ and 7.9–16.2 × 10^−3^ min^−1^. It can be observed that the parameters of the kinetic constants for both series of samples increase with increasing Ag content, and this phenomenon was observed at both wavelengths of light. The results indicate that the Ag content greatly influences the photodegrading rate (k_cal_) of the silver-modified g-CN bulk and nanosheet samples. The highest photodegrading rate was estimated for all silver-modified g-CN bulk and nanosheet materials. Under UV light, both silver-modified g-CN series showed similar values of kinetic constants in the range 7.0–8.8 × 10^−3^ min^−1^. Under VIS irradiation, the silver-modified g-CN series exhibit kinetic constants in the range of 9.5–16.2 × 10^−3^ min^−1^. The g-CN-exf-1.5%Ag sample achieved the highest kinetic constant values (16.2 × 10^−3^ min^−1^) and considerable photodegradation activity (98%) after 3 h of exposure to visible light towards AO7.

The enhanced photocatalytic performance of g-CN bulk and nanosheet materials modified with Ag NPs towards AO7 under UV and VIS light can be explained as follows. Various studies have reported that ∙O_2_^−^ is the dominant reactive species during photocatalytic reactions under UV/VIS for pristine materials based on g-CN-JP, g-CN-JP-150, g-CN-exf, and g-CN-exf-150^26,82,83^. The addition of silver (g-CN-exf-x% Ag and g-CN-JP-x% Ag) positively affected the photoactivity of both materials under UV or VIS irradiation^26,82,83^. When the Fermi level of silver is decreased relative to the conduction band energy of graphitic carbon nitride (g-C_3_N_4_), photoexcited electrons in the g-C_3_N_4_ conduction band are generated. These electrons subsequently react with oxygen on silver nanoparticles to produce superoxide radicals. This phenomenon becomes more pronounced as the quantity of silver nanoparticles in the composites increases^84^. Ag nanoparticles enhance photocatalytic activity, especially under visible light, by improving the separation of photogenerated electrons and holes in composites. Both Ag and g-C_3_N_4_ nanoparticles absorb visible light and generate electrons and holes. Ag nanoparticles' electrons are induced by visible light, while the holes are located on the Ag nanoparticles. The electrons generated by g-C_3_N_4_ combine with the plasmon-induced holes formed by the absorption of plasmons by the Ag nanoparticles. However, the holes in the valence band remain on g-C_3_N_4_, which oxidizes organic pollutants under visible light^85,86^. Subsequently, the ∙O_2_^−^ radicals decompose AO7 to phenol, rapidly decomposing to 1,4-benzoquinone, water, and CO_2_^87,88^.

The increasing Ag content in the composites then forms more ∙O_2_^−^ radicals in UV light^26,89^. The photodegradation of AO7 to phenol in UV is slightly less effective, and the formation of reaction products, 1,4-benzoquinone, and subsequently water and CO_2_ from phenol, is well described in these studies^87,88,90^.

## Conclusions

We have successfully synthesized surface-modified g-CN bulk and nanosheet materials by introducing silver nanoparticles and examined their effectiveness as photocatalysts in the degradation of AO7 dye molecules in water systems. Our study compared the photodegradation performances of two lamps with wavelengths of 368 nm (UV light) and 420 nm (VIS light), respectively. The results of this study can be summarized as follows.

- The presence of silver nanoparticles was confirmed through SEM and TEM analyses.
- During the evaluation of the particle size distribution of Ag, an interesting effect was observed for the g-CN bulk materials. It was noticed that as the Ag content increased from 0.5 to 3 wt%, the average diameter of the Ag nanoparticles decreased from 8.1 ± 1.9 nm to 5.2 ± 1.6 nm.
- The photodegradation activity of the samples was observed to increase with an increase in the silver content. The best UV photocatalytic performance was achieved for both surfaces modified with silver nanoparticles g-CN materials, specifically those with the highest amount of 3 wt% Ag. These materials exhibited more than 75% and 78% efficiency, respectively.
- In both series of materials, the photocatalytic activity produced by visible light was more significant than the activity generated by ultraviolet light. The bulk and nanosheet g-CN materials containing 1.5 wt% Ag displayed the highest activity, with rates of 92% and 98%, respectively.

This study introduces a novel method for creating photocatalysts through the modification of g-CN materials by incorporating Ag nanoparticles. The Ag NPs are added at concentrations of 0.5%, 1.5%, and 3% using a wet and low-temperature chemical process that is both cost-effective and environmentally friendly. The resulting photocatalysts have demonstrated the ability to degrade organic pollutants, which could have practical applications in wastewater treatment.

### Supplementary Information

Supplementary Information.

## Data Availability

The data will be available on the request from the corresponding author (Dr. Monika Michalska, monika.kinga.michalska@gmail.com).
