# Supplementary material for: Comparative study of photocatalysis with bulk and nanosheet graphitic carbon nitrides enhanced with silver
Source: Sci Rep. 2024 May 20;14:11512. doi: 10.1038/s41598-024-62291-w (PMC11106318; doi:10.1038/s41598-024-62291-w)
Supplement: Supplementary file 1 — Supplementary Information. [file 41598_2024_62291_MOESM1_ESM.pdf]

Supplementary Material

Comparative study of photocatalysis with bulk and nanosheet graphitic carbon nitrides enhanced with silver

Monika Michalska[1]*, Jiri Pavlovsky[1], Grazyna Simha Martynkova[2], Gabriela Kratosova[2], Viktoria Hornok[3], Peter B. Nagy[3], Vlastimil Novak[1], Tamas Szabo[3]

[1]Department of Chemistry and Physico-Chemical Processes, Faculty of Materials Science and Technology, VSB-Technical University of Ostrava, 17. listopadu 2172/15, 708 00 Ostrava-Poruba, Czech Republic
[2]Nanotechnology Centre, CEET, VSB-Technical University of Ostrava, 17. listopadu 2172/15, 708 00, Ostrava-Poruba, Czech Republic
[3]Department of Physical Chemistry and Materials Science, University of Szeged, Rerrich Béla Tér. 1, H-6720 Szeged, Hungary

*Corresponding author: monika.kinga.michalska@gmail.com (Dr. M. Michalska)

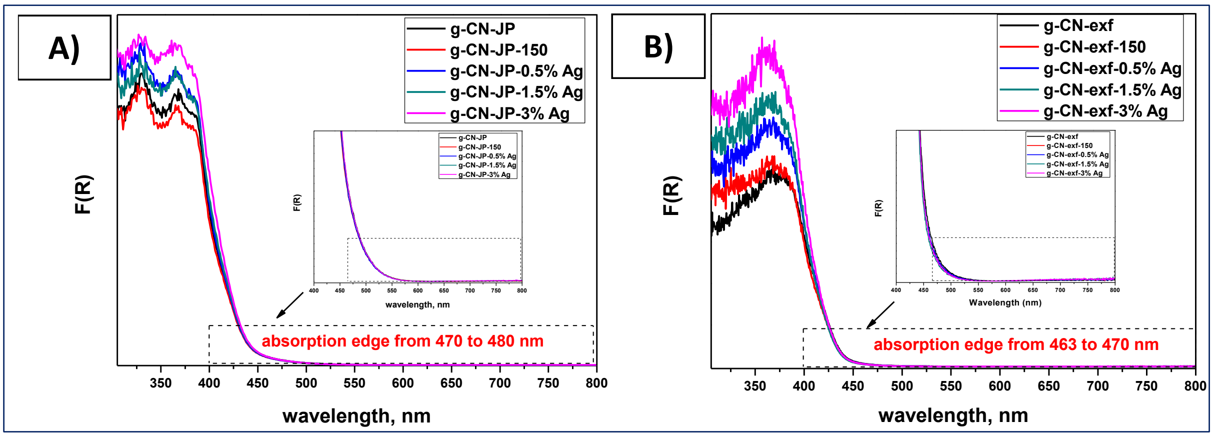

Fig. S1. UV-Vis DRS absorption spectra of silver-modified g-CN bulk (**A**), and nanosheet (**B**) materials.

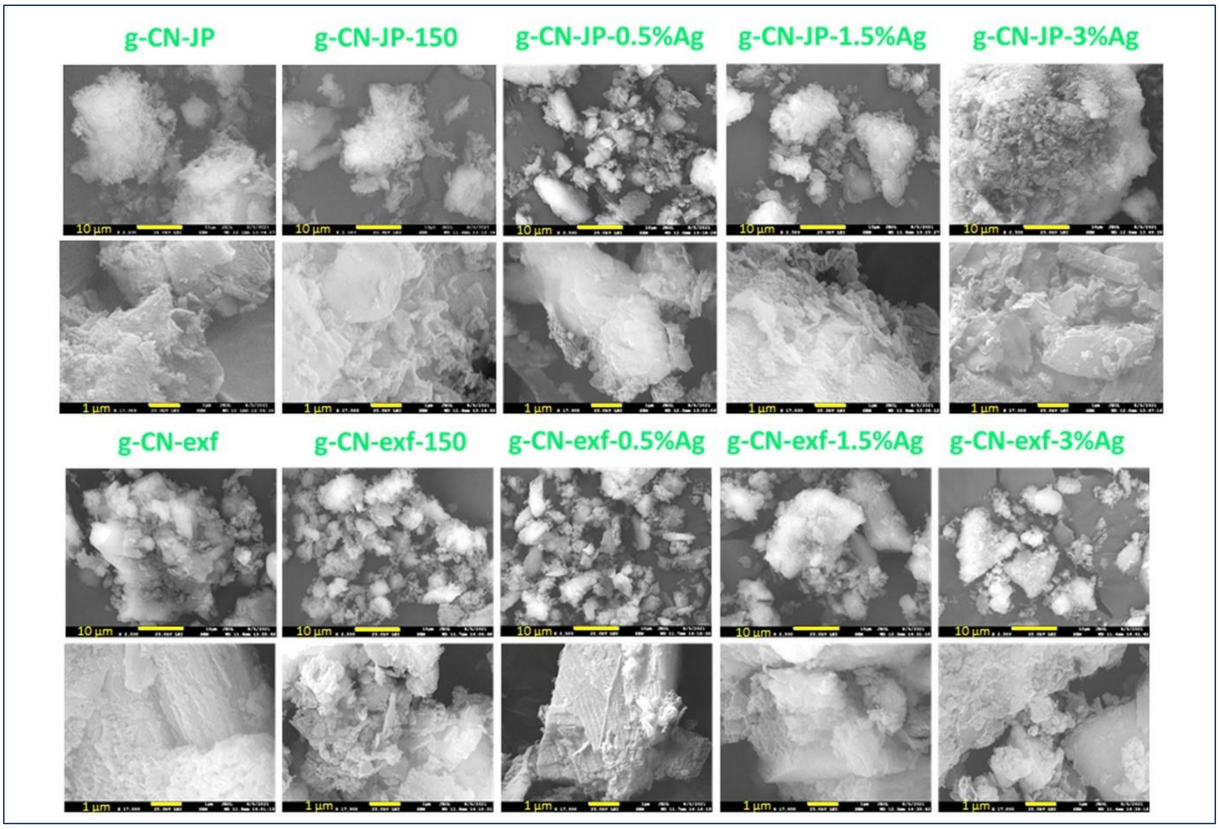

Fig. S2. SEM images of silver-modified g-CN bulk and nanosheet materials at two magnifications: 2500x (scale bar 10 µm) and 17000x (scale bar 1 µm).

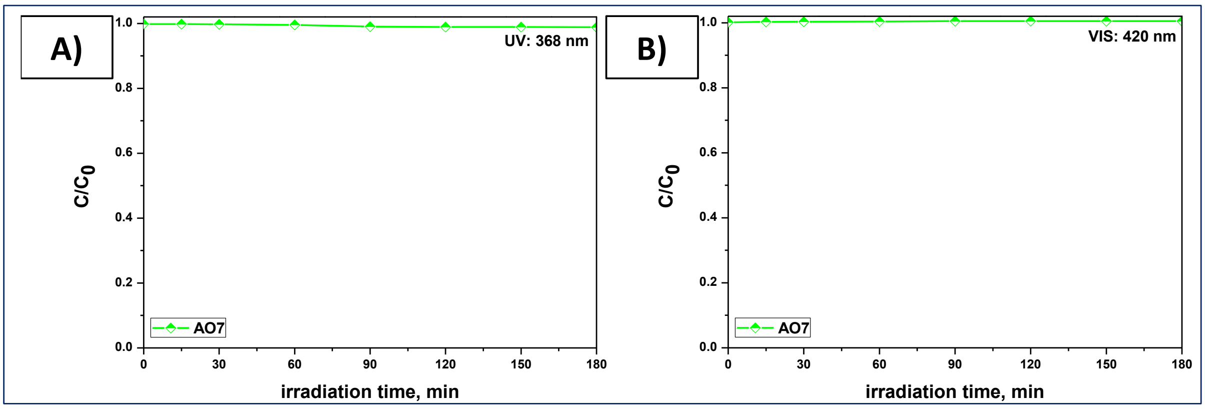

Fig. S3. Photocatalytic degradation C/C_0 ratio as a function of the irradiation time for AO7 dye under (**A**) UV (368 nm), and (**B**) VIS (420 nm) lamps.

Table S1. A literature survey of graphitic carbon nitride (g-C_3N_4, g-CN) composites with silver nanoparticles utilized for photodegradation processes over selected dyes.

g-CN modified with Ag	Dye	Irradiation: time, light	Photodegradation activity, %	Ref.
Ag/g-C_3N_4<br>Ag: 0.1 to 5 wt%	MO	1 h, visible-light irradiation (400 nm < λ < 680 nm)	92% (Ag(5)/g-C_3N_4),<br>91% (Ag(2)/g-C_3N_4)	[1]
3%-Ag/g-C_3N_4	RhB	100 min, simulated sunlight irradiation, which was obtained from a 500 W Xe lamp	almost 100%	[2]
Ag@*g*-C_3N_4 NSs (3 mM)	MB,<br>RhB	210 min MB visible-light irradiation<br>250 min RhB visible-light irradiation	~100% MB degradation<br>~89% of RhB degradation	[3]
g/g-C_3N_4 (1:2)	MO	1 h VIS 420 nm	10%	[4]
Ag/g-C_3N_4	MO	1 h VIS 420 nm	90%	[5]
Ag/g-CN	MO	1 h VIS 420 nm	38%	[6]
1.0%Ag/g-C_3N_4	MO	1 h VIS 420 nm	50%	[7]
g-CN-JP-0.5%Ag<br>g-CN-JP-1.5%Ag<br>g-CN-JP-3%Ag	AO7	3 h UV 368 nm	65%<br>68%<br>75%	This work
g-CN-JP-0.5%Ag<br>g-CN-JP-1.5%Ag<br>g-CN-JP-3%Ag	AO7	3 h VIS 420 nm	91%<br>92%<br>92%	This work
g-CN-exf-0.5%Ag<br>g-CN-exf-1.5%Ag<br>g-CN-exf-3%Ag	AO7	3 h UV 368 nm	66%<br>75%<br>78%	This work
g-CN-exf-0.5%Ag<br>g-CN-exf-1.5%Ag<br>g-CN-exf-3%Ag	AO7	3 h VIS 420 nm	95%<br>98%<br>96%	This work

MO – methyl orange, rhodamine B – RhB, methylene blue - MB, acid orange 7 – AO7.

References:

1. Y. Yang, Y. Guo, F. Liu, X. Yuan, Y. Guo, S. Zhang, W. Guo, M. Huo, Preparation and enhanced visible-light photocatalytic activity of silver deposited graphitic carbon nitride plasmonic photocatalyst, Appl. Catal. B-Environ. 142-143 (2013) 828–837.
2. K. Qi, Y. Li, Y. Xie, S.-Y. Liu, K. Zheng, Z. Chen, R. Wang, Ag loading enhanced photocatalytic activity of g-C_3N_4 porous nanosheets for decomposition of organic pollutants, Front. Chem. 7 (2019) 91.
3. M.E. Khan, T.H. Han, M.M. Khan, M.R. Karim, M.H. Cho, Environmentally sustainable fabrication of Ag@g-C_3N_4 nanostructures and their multifunctional efficacy as antibacterial agents and Photocatalysts, ACS Appl. Mater. 1 (2018) 2912–2922.

4. Y. Chen, W. Huang, D. He, Y. Situ, H. Huang, Construction of heterostructured g-C_3N_4/Ag/TiO_2 microspheres with enhanced photocatalysis performance under visible-light irradiation, *ACS Appl. Mater. Interfaces* 6 (2014) 14405–14414.
5. S. Yan, Z.S. Li, Z.G. Zou Photodegradation performance of g-C_3N_4 Fabricated by directly heating melamine, Langmuir 25 (2009) 10397–10401.
6. R. Liu, W. Yang, G. He, W. Zheng, M. Li, W. Tao, M. Tian, Ag-modified g-C_3N_4 prepared by a one-step calcination method for enhanced catalytic efficiency and Stability, *ACS Omega* 5 (2020) 19615–19624.
7. L. Ge, *C. Han, J. Liu, Y. Lee,* Enhanced visible light photocatalytic activity of novel polymeric g-C_3N_4 loaded with Ag nanoparticles, *Appl. Catal. A: General* 409–410 (2011) 215–222.
